# Left Inferior Frontal Activations Depending on the Canonicity Determined by the Argument Structures of Ditransitive Sentences: An MEG Study

**DOI:** 10.1371/journal.pone.0037192

**Published:** 2012-05-22

**Authors:** Tomoo Inubushi, Kazuki Iijima, Masatoshi Koizumi, Kuniyoshi L. Sakai

**Affiliations:** 1 Department of Basic Science, Graduate School of Arts and Sciences, The University of Tokyo, Komaba, Meguro-ku, Tokyo, Japan; 2 CREST, Japan Science and Technology Agency, Sanban-cho, Chiyoda-ku, Tokyo, Japan; 3 Japan Society for the Promotion of Science, Honmachi, Kawaguchi-shi, Saitama, Japan; 4 Department of Linguistics, Tohoku University, Kawauchi, Aoba-ku, Sendai-shi, Miyagi, Japan; University of Leicester, United Kingdom

## Abstract

To elucidate the relationships between syntactic and semantic processes, one interesting question is how *syntactic* structures are constructed by the argument structure of a verb, where each argument corresponds to a *semantic* role of each noun phrase (NP). Here we examined the effects of *possessivity* [sentences with or without a possessor] and *canonicity* [canonical or noncanonical word orders] using Japanese ditransitive sentences. During a syntactic decision task, the syntactic structure of each sentence would be constructed in an incremental manner based on the predicted argument structure of the ditransitive verb in a verb-final construction. Using magnetoencephalography, we found a significant canonicity effect on the current density in the left inferior frontal gyrus (IFG) at 530–550 ms after the verb onset. This effect was selective to canonical sentences, and significant even when the precedent NP was physically identical. We suggest that the predictive effects associated with syntactic processing became larger for canonical sentences, where the NPs and verb were merged with a minimum structural distance, leading to the left IFG activations. For monotransitive and intransitive verbs, in which structural computation of the sentences was simpler than that of ditransitive sentences, we observed a significant effect selective to noncanonical sentences in the temporoparietal regions during 480–670 ms. This effect probably reflects difficulty in semantic processing of noncanonical sentences. These results demonstrate that the left IFG plays a predictive role in syntactic processing, which depends on the canonicity determined by argument structures, whereas other temporoparietal regions would subserve more semantic aspects of sentence processing.

## Introduction

The ability to embed phrases within phrases and to construct hierarchical sentence structures has been proposed to be a fundamental property of language faculty that is unique to humans [1]. This ability based on syntactic knowledge enables humans to utilize the expressive and creative power of language. Recent functional magnetic resonance imaging (fMRI) studies have shown that processing syntactic structures of sentences significantly elicits localized activation in the brain. To contrast sentences with canonical (i.e., typical) and noncanonical word orders has been one effective paradigm for further elucidating syntactic processes [2]–[6]. Using sentences in German, Hebrew, and Japanese, larger responses to sentences with noncanonical word orders have been reported in some cortical regions including the left inferior frontal gyrus (IFG), left lateral premotor cortex, and left posterior superior/middle temporal gyrus (pSTG/MTG). Recent lesion studies have also demonstrated that patients with a lesion in the left IFG showed profound deficits in the comprehension of noncanonical sentences [7], [8]. On the other hand, it has been reported that the change in word orders for the animacy of arguments affects the left IFG activation [9]. It should be then clarified how the syntactic processes of sentences are actually influenced by the animacy itself, together with any other semantic factors that may affect syntactic features (e.g., possessor/benefactive). To elucidate such underlying syntactic and semantic processes, we chose *ditransitive* sentences, each of which included a verb, as well as dative and accusative noun phrases (NPs). The syntactic structures of a ditransitive sentence can be partially determined by the argument structure of the verb, where two arguments correspond to different semantic roles of the NPs. Here we used magnetoencephalography (MEG) to examine more detailed temporal aspects of cortical activity.

It is interesting to note that English ditransitive sentences can be divided into two types: double object sentences (1a) and prepositional dative sentences (1b) [10].

(1a) *I threw John the ball*

(1b) *I threw the ball to John*

These two types of sentences have different argument structures of the verb (*threw* in this example), which result in different sentence meanings [11], [12]. The argument structure of a double object sentence is [agent, possessive goal, theme], representing “*X* (agent) causes *Y* (possessive goal) to have *Z* (theme)”. *Y* is the goal to which *Z* goes as the result of its movement or transfer, and at the same time *Y* should become the possessor of *Z*; *Y* is thus defined as a possessive goal. On the other hand, the argument structure of a prepositional dative sentence is [agent, locative goal, theme], representing “*X* (agent) causes *Z* (theme) to go to *Y* (locative goal)”. Here, *Y* is simply the goal to which *Z* goes as the result of its movement or transfer; *Y* is thus called a locative goal. With such a different semantic role, a possessive goal has an additional privilege to have the property of the *possessor*. In summary, a possessive goal defines a sentence with a possessor (P^+^) like (1a), whereas a locative goal defines a sentence without a possessor (P^–^) like (1b). This point becomes clearer in the following examples.

(2a) *I threw the target the ball (An asterisk denotes an ungrammatical sentence.)

(2b) *I threw the ball to the target*

As shown in (2a, b), the inanimate noun (*the target* in this example) that cannot be a possessor makes the sentence ungrammatical in double object sentences, whereas it is allowed in prepositional dative sentences.

Another critical factor regarding the argument structures of ditransitive sentences is the order of two NPs.

(3a) *I threw the ball John

(3b) *I threw to John the ball

As shown in (3a, b) where (3b) has no heavy NP [13], scrambling the word order is not allowed in English, even if these sentences preserve the argument structures in (1a, b). One relevant hypothesis on the preference of word orders in general is the linearization of a grammatical feature or order-related factors. For example, a hierarchy of subject > direct object > indirect object > oblique (other) object (from highest to lowest), that of nominative > dative > accusative, and that of animate > inanimate have been proposed in cross-linguistic studies [14]. Previous fMRI studies contrasting canonical and noncanonical sentences have interpreted that an activation increase at the left IFG was due to the violation of these linearization rules [3], [9]. However, any theories based on such linearization alone fail to explain the word orders of (1a, b), because in (1a), an indirect object (*John*) precedes a direct object (*the ball*), while in (1b), an accusative and inanimate object (*the ball*) precedes a dative and animate object (*to John*). An alternative approach is a structural model that focuses on the syntactic structures of sentences. This model predicts that the examples of (1a, b) actually have different syntactic structures, such that the possessor/benefactive (*John*) in (1a) takes the higher position than the theme (*the ball*), while the theme (*the ball*) in (1b) is higher than the prepositional phrase (*to John*) [13], [15]. Therefore, we may naturally assume that the basic structures of P^+^ and P^–^ sentences are also different in languages other than English.

One notable difficulty here is to separate the factor of word order from the grammaticality of sentences. This problem can be resolved by using other natural languages, in which the basic features of the argument structures are universal, but scrambling is allowed. Indeed, the argument structures of Japanese ditransitive verbs are either [agent, possessive goal, theme] or [agent, locative goal, theme], where each argument is marked by nominative (Nom), dative (Dat), or accusative (Acc) case marker. Note that the dative case particle ‘-*ni*’ is used for both sentence types [16], and that an agent can be a phonetically null subject (pro-drop) in Japanese, as well as in Spanish and Italian [17].

(4a) *‘yuujin-ni kagu-o ageta’*

(a word-by-word translation in English: *friend*-Dat *furniture*-Acc *gave*)

*Someone gave his friend furniture*

(4b) ‘kagu-o nikai-ni ageta’

(*furniture*-Acc *upper floor*-Dat *lifted*)

*Someone lifted furniture to the upper floor*

These two sentences are actually paired, sharing the same accusative NP (theme) and phonologically same verb (‘*ageta*’), but having different meanings and argument structures. Such ditransitive verb pairs actually form a general class of verbs, just like the English verb *threw* in (1a, b). We used a set of sentence stimuli (Table 1), in which each of the animate dative NPs is naturally interpreted as a possessive goal that defines a P^+^ sentence like (4a), whereas each of the inanimate dative NPs is naturally interpreted as a locative goal that defines a P^–^ sentence like (4b). This is substantiated by the fact that English P^+^ sentences become odd in meaning with addition of a sentence that implies failure of transfer: e.g., *My aunt gave my brother some money for new skis, but he never got it [18], which is also true for Japanese P^+^ sentences: e.g., ‘*yuujin-ni kagu-o ageta-ga, sono yuujin-wa moratte inakatta’ (*Someone gave his friend furniture, but his friend never got it). Therefore, native speakers of Japanese can correctly differentiate two meanings of the phonologically same verb. The argument structure of a verb in a grammatical sentence can be thus determined from the animacy of the dative NP with ‘*-ni*’ and the presence of the accusative NP with ‘-*o*’ (theme) in each sentence, which are all given before the verb presentation. Because scrambling is allowed in Japanese, the sentences with dative before accusative (DA) order and those with accusative before dative (AD) order are all grammatical with same meanings (Table 2). By using these four separate conditions, we can examine the effect of *possessivity* (P^+^ or P^–^) and that of word orders without changing the grammaticality of the sentences.

**Table 1:** Examples of ditransitive sentences used in the present study.

Sentence with a possessor (P^+^)	Sentence without a possessor (P^–^)
‘*yuujin-ni kagu-o ageta*’	‘*kagu-o nikai-ni ageta*’
*Someone gave his friend furniture*	*Someone lifted furniture to the upper floor*
‘*jouren-ni sushi-o dashita*’	‘*sushi-o syokutaku-ni dashita*’†
*Someone served a regular customer sushi*	*Someone placed sushi on the table*
‘*ooya*-*ni yachin-o ireta*’	‘*yachin-o kinko*-*ni ireta*’
*Someone paid the owner the house rent*	*Someone put the house rent into the safe*
‘*chijin-ni shinsya-o kaeshita*’	‘*shinsya-o syako*-*ni kaeshita*’
*Someone returned an acquaintance his new car*	*Someone returned his new car to the garage*
‘*joukyaku-ni kippu-o modoshita*’	‘*kippu-o saifu-ni modoshita*’
*Someone returned the passenger the ticket*	*Someone returned the ticket to the wallet*
‘*gyousya-ni kinzoku-o nagashita*’	‘*kinzoku-o igata*-*ni nagashita*’
*Someone sent the trader the metal*	*Someone poured metal into the mold*
‘*shinseki-ni kozutsumi-o okutta*’	‘*kozutsumi-o yashiki*-*ni okutta*’
*Someone sent his relative the gift*	*Someone sent the gift to the residence*
‘*kanja-ni yakuhin*-*o todoketa*’	‘*yakuhin-o byouin-ni todoketa*’
*Someone sent the patient the drugs*	*Someone delivered the drugs to the hospital*
‘*zen’in*-*ni soubi-o tsuketa*’	‘*soubi-o kabegiwa-ni tsuketa*’
*Someone gave everyone the equipment*	*Someone attached the equipment to the wall*
‘*shinzoku*-*ni zaisan-o utsushita*’	‘*zaisan-o chika-ni utsushita*’
*Someone sent his relative property*	*Someone delivered property to the basement*
‘*suifu-ni kobune-o watashita*’	‘*kobune-o taigan-ni watashita*’
*Someone gave the sailor a boat*	*Someone moved a boat to the opposite shore*
‘*kouhai-ni furuhon-o yatta*’	‘*furuhon-o katasumi-ni yatta*’
*Someone gave the junior fellow a used book*	*Someone put a used book in the corner*
‘*sakusya-ni tegami-o yoseta*’	‘*tegami-o madogiwa-ni yoseta*’
*Someone sent the author letters*	*Someone put letters near the window*

The argument structures of the verbs in the P^+^ and P^–^ sentences are [agent, possessive goal, theme] and [agent, locative goal, theme], respectively. We omitted an agent from the stimuli, as a phonetically null subject (pro-drop) is allowed in Japanese. For each pair of P^+^ and P^–^ sentences, as shown in each line of the Table, the same accusative NP and phonologically same verb were used. All used verbs, 26 of 100 dative NPs (always animate for P^+^ and inanimate for P^–^), and 13 of 50 accusative NPs (always inanimate), are shown here in the alphabetical order of Japanese verbs. †Some P^–^ sentences might imply the presence of a recipient, but an inanimate dative NP itself cannot become a possessor for all examples.

**Figure 1:**
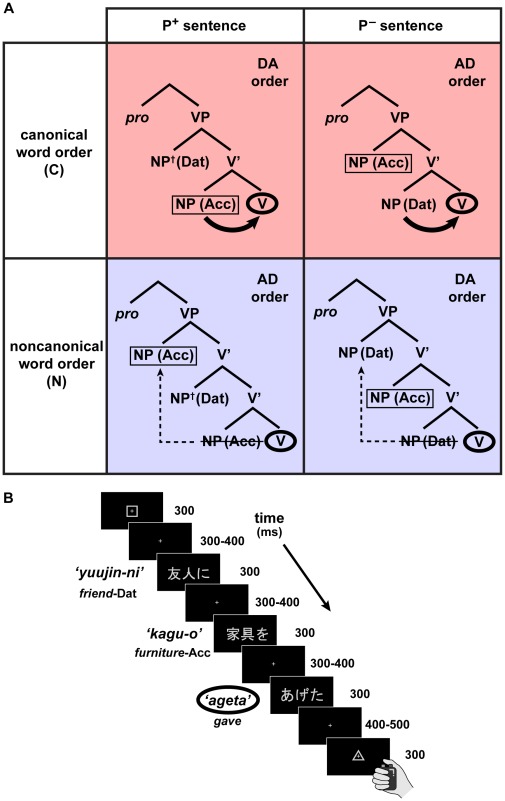
Structures of ditransitive sentences, together with serial presentation of each sentence. (A) A succinct version of linguistic tree structures representing the syntactic structures of ditransitive sentences. P^+^ and P^–^ sentences are in columns, while canonical (C, shown in red) and noncanonical (N, shown in blue) word orders, i.e., the canonicity of sentences, are in rows. Dat, dative case marker; Acc, accusative case marker; pro, pronoun, which is a phonetically null subject. For the syntactic structures of noncanonical sentences (lower row), a noun phrase (NP) closest to a verb (V) is moved to the front of another NP (dashed arrow), and merged with the higher V-bar (V’) to form a verb phrase (VP). The moved NP then leaves a *trace* in its original or canonical position, producing a gap with a longer structural distance between the second NP and V. In our paradigm, each pair of P^+^ and P^–^ sentences had the same accusative NP (boxed) and phonologically same verb (circled) (see Table 1). We examined the predictive effects of precedent NPs on the verb, which were expected to be larger for the *canonical* sentences with shorter structural distances (curved arrows) than the noncanonical sentences. Among the four conditions, an animate NP (with a dagger) appeared only as the dative NP of the P^+^ sentences. (B) A single trial with a ditransitive sentence. A grey square was presented to inform the participant that the trial had begun. Next, a sentence, consisting of two NPs and a verb, was presented in a serial, phrase-by-phrase manner. A grey triangle was shown after a verb to inform participants to initiate a button press. Interstimulus intervals were randomly varied so that the responses to verbs were not confounded with those to precedent NPs. We mainly analyzed the cortical responses to ditransitive verbs.

There are two possible syntactic structures for each of Japanese ditransitive sentences: either canonical (C) or noncanonical (N) in word order (Figure 1A). We regard *canonicity* (canonical or noncanonical word orders) as another key concept in our present study. Canonicity involves structural computation, in that a long-distance dependency (e.g., an NP-movement) is necessary to yield the surface word order of noncanonical sentences. According to current linguistic theories [19], [20], the second NP and verb in a canonical sentence are merged to form a V-bar (V’) with a minimum *structural distance* (the upper panels in Figure 1A). The first NP and V’ are then merged to form a verb phrase (VP). On the other hand, in a noncanonical sentence, the second NP and lower V’ are merged to form a higher V’ (the lower panels in Figure 1A). The first NP and higher V’ are then merged to form a verb phrase (VP), making a longer structural distance between the verb and each of the first and second NPs. According to linguistic theories on the Japanese language [21]–[26], the canonical word order of P^+^ sentences is DA. Although it has been controversial whether the canonical word order of P^–^ sentences is AD or DA [23], [27], a recent behavioral study has indicated that the P^–^ sentences with the AD order were produced more often than the P^+^ sentences with the AD order [28]. We hypothesize that the P^–^ sentences with the AD order, as well as the P^+^ sentences with the DA order, are canonical in word order. The differential *canonical* word orders in Japanese ditransitive sentences (sentence examples 4a, 4b), depending on the semantic contrasts between P^+^ and P^–^ sentences, are also consistent with the *grammatical* word orders in English ditransitive sentences (sentence examples 1a, 2b), suggesting the universal property of syntactic processes, which are indeed influenced by the accompanying semantic processes. In the analyses of behavioral and MEG data, we performed two-way repeated measures analysis of variance (rANOVA) (factors: possessivity × canonicity), and particularly focused on the main effect of canonicity, rather than on a single condition out of the four conditions, because the linearization of order related-factors (e.g., direct object > indirect object, dative > accusative, and animate > inanimate) can be canceled out between P^+^ and P^–^ sentences, leaving out the canonicity effects alone. Here we mainly analyzed the cortical responses to ditransitive verbs.

Some previous fMRI studies have examined the effects of verb argument structures or those of verb groups with different syntactic phrase types, and have reported activation in the left IFG, as well as in the temporoparietal regions [29], [30]. However, these previous studies have used a lexical decision or semantic decision task that involves syntactic factors only implicitly, and it has been already known that the cortical activation depends on the choice of linguistic tasks, even if target stimuli are kept identical [31], [32]. In previous fMRI and MEG studies, we have clearly shown that selective activations are observed in the left IFG during explicit syntactic processing (in a syntactic decision task), when compared with implicit syntactic processing (in semantic decision and other tasks) [33], [34]. It was thus necessary to use an explicit syntactic decision task in the present study (Figure 1B), together with well-controlled stimuli as shown in Table 1. It is thus expected that such an explicit grammaticality judgment selectively activates the left IFG.

There are at least two factors that can differentiate processing of canonical vs. noncanonical sentences. One factor is certain processing loads, which become larger for *noncanonical* sentences (i.e., N > C) as indicated by behavioral studies [35]–[38]. Previous neuroimaging studies have also suggested that the activation of the left IFG, as well as that of the left temporoparietal regions, may reflect the load of the short-term memory [4], the computation of movement (i.e., the displacement of words) [39], or the syntactic processing associated with noncanonical word orders [6], [8]. The other factor is the predictive effect, which would become larger for *canonical* sentences (i.e., C > N). Recently, some neuroimaging studies have shown that the left IFG activation reflects predictive effects associated with syntactic processing [33], [40]. For example, in our previous MEG study, we showed that the left IFG responses to a transitive verb in an object-verb sentence were enhanced, selectively for syntactic judgments on minimum sentences consisting of an NP and a verb [33]. Because this enhancement was observed irrespective of syntactic anomaly itself, it cannot be explained by computations of matching or error detection/correction alone. The left IFG responses may reflect more specific computations or predictive effects, such that an accusative NP predicts a next-coming verb as *transitive* verb, which is the only possible verb type for minimum sentences. Moreover, this previous result cannot be explained by associative memory or transition probability, and it provides an explicit hypothesis, such that a precedent NP *facilitates* syntactic processing when the NP and verb are merged with a minimum structural distance. Indeed, modern linguistics has elucidated the importance of minimizing structural distance [41], and we hypothesize that the predictive processing can be regarded as a function of structural distance. In terms of on-line computations, canonicity and predictability are closely related in syntactic processing, because a structural distance becomes minimum for canonical sentences. If the syntactic structure of a canonical sentence is readily predicted in an incremental manner (see Figure 1A), it is likely that the sentence becomes easier to comprehend for the participants. Therefore, we expected to observe any predictive effects on a ditransitive verb for *canonical* sentences. To detect activation changes in an unbiased manner, we adopted whole-brain analyses. We had focused on an earlier period of 100–300 ms for a minimum sentence in the previous MEG study, but it is expected that the predictive effect becomes delayed when the two NPs and verb are merged in more complex ditransitive sentences. We thus extended the time window as late as 700 ms, which was the stimulus interval plus the following shortest interstimulus interval (ISI) (Figure 1B), to search for any C > N or N > C effects in the present study.

## Materials and Methods

### Participants

The participants in the present study were 11 native Japanese speakers. One participant, whose data contained a large amount of noise due to eye movement or blinking, was discarded from the analysis (the ratio of trials with a noise >2500 fT over the period of –100–700 ms after the verb onset: 62.2% for the excluded participant and 9.7–27.0% for the others), leaving a total of 10 participants (21–32 years; 1 female). The 10 participants showed right-handedness (laterality quotients: 50–100) as determined by the Edinburgh inventory [42]. Prior to participation in the study, written informed consent was obtained from all participants after the nature and possible consequences of the studies were explained. Approval for these experiments was obtained from the institutional review board of the University of Tokyo, Komaba.

### Stimuli

In our paradigm, we prepared 50 grammatical sentences under each of four conditions (Table 2). Each sentence consisted of a dative NP (always animate for P^+^ and inanimate for P^–^), an accusative NP (always inanimate), and a verb. Although many ditransitive verbs take either P^+^ or P^–^ alone, we used here such verbs that can take both P^+^ and P^–^. There has been no assessment of whether Japanese ditransitive verbs were preferentially associated with P^+^ or P^–^ structures, but some English ditransitive verbs have been assessed whether they were preferentially associated with double object sentences or prepositional dative sentences [43]. For each pair of P^+^ and P^–^ sentences, as shown in each line of Table 1, the same verb and same accusative NP were used to control the stimuli among different conditions. All verbs and NPs always consisted of three letters of kana (or katakana) and kanji to ensure a consistent reading time. We prepared 200 original sentences (50×4) that were all grammatical. Each sentence stimulus appeared only twice for each participant.

**Table 2:** Examples of ditransitive sentences under the four conditions.

Possessivity	Canonicity	Example
P^+^ sentences	Canonical	‘*yuujin-ni kagu-o ageta*’ (*friend*-Dat *furniture*-Acc *gave*)
		*Someone gave his friend furniture*
	Noncanonical	‘*kagu-o yuujin-ni ageta*’ (*furniture*-Acc *friend*-Dat *gave*)
		*Someone gave his friend furniture*
P^–^ sentences	Canonical	‘*kagu-o nikai-ni ageta*’ (*furniture*-Acc *upper floor*-Dat *lifted*)
		*Someone lifted furniture to the upper floor*
	Noncanonical	‘*nikai-ni kagu-o ageta*’ (*upper floor*-Dat *furniture*-Acc *lifted*)
		*Someone lifted furniture to the upper floor*

Dat, dative case marker; Acc, accusative case marker. A word-by-word translation in English is shown after each example. In Japanese, the sentences with dative before accusative (DA) order and those with accusative before dative (AD) order are all grammatical and commonly used. We hypothesize that “the P^+^ sentences with the DA order” and “the P^–^ sentences with the AD order” are canonical in word order; the canonicity depends on the semantic contrasts between P^+^ and P^–^ sentences (see the Introduction).

To examine transition probabilities between words, all of our ditransitive sentences used in the present study were checked against actual examples of Japanese sentences on the internet, searched with *Google* (http://www.google.co.jp/) and *Yahoo* (http://www.yahoo.co.jp/). Regarding the transition probabilities from the second NP to the verb, a one-way rANOVA showed no significant difference among animate dative NPs, inanimate dative NPs, and accusative NPs used as second NPs [*F* (2, 98) = 0.15, *p* = 0.85]. We also compared the transition probabilities from the two NPs to the verb among the four conditions; two-way ANOVAs (possessivity × canonicity) showed no significant main effects of possessivity [*F* (1, 155) = 0.88, *p* = 0.35] and canonicity [*F* (1, 155) = 0.85, *p* = 0.36], with no significant interaction ([*F* (1, 155) = 0.40, *p* = 0.53].

For a syntactic decision task, we added grammatical and ungrammatical modified sentences (120 each) to the grammatical ditransitive sentences. To ensure that the participants paid attention to the relationships between both NPs and a verb, two sets of modified sentences were prepared by changing the ditransitive verbs of the subset of original sentences into either monotransitive verbs (compatible with an accusative NP) or intransitive verbs (compatible with a dative NP) (see Table 3). One set of modified sentences (60 each for grammatical and ungrammatical modified sentences) had *monotransitive* verbs that cannot take a dative NP. The grammaticality was thus dependent on the presence of a dative NP. For example, ‘*yuujin-ni kagu-o ageta*’ and ‘*nikai-ni kagu-o ageta*’ (see sentence examples 4a, 4b) were changed to ‘*yuujin-ni kagu-o migaita’ (*friend*-Dat *furniture*-Acc *polished*) and ‘*nikai-ni kagu-o migaita’ (*upper floor*-Dat *furniture*-Acc *polished*), respectively. From the ungrammatical sentences with the animate dative NPs, grammatical sentences were made by changing the dative case particle (‘-*ni*’) to the nominative case particle (‘*-ga*’) (Table 3). From the ungrammatical sentences with the inanimate dative NPs, grammatical sentences were made by changing the dative case particle (‘-*ni*’) to the locative postposition (‘*-de*’).

**Table 3:** Examples of grammatical modified sentences with either monotransitive or intransitive verbs.

Verb type	Example
Monotransitive	‘*yuujin-ga kagu-o migaita*’ (*friend*†-Nom *furniture*-Acc *polished*)
	*His friend polished furniture*
	‘*nikai-de kagu-o migaita*’ (*upper floor*-Loc *furniture*-Acc *polished*)
	*Someone polished furniture at the upper floor*
Intransitive	‘*kagu-ga yuujin-ni tsuita*’ (*furniture*-Nom *friend*†-Dat *arrived*)
	*A piece of furniture arrived at his friend*
	‘*kagu-ga nikai-ni tsuita*’ (*furniture*-Nom *upper floor*-Dat *arrived*)
	*A piece of furniture arrived at the upper floor*

Nom, nominative case marker; Loc, locative postposition. Canonical sentences are shown here. Noncanonical sentences were made by scrambling two NPs in each sentence. Among these conditions, an animate NP (with a dagger) appeared either as the nominative NP or as the dative NP.

Another set of modified sentences (60 each for grammatical and ungrammatical modified sentences) had *intransitive* verbs that cannot take an accusative NP. The grammaticality was thus dependent on the presence of an accusative NP. For example, we prepared ‘*kagu-o yuujin-ni tsuita’ (*furniture*-Acc *friend*-Dat *arrived*) and ‘*kagu-o nikai-ni tsuita’ (*furniture*-Acc *upper floor*-Dat *arrived*). Grammatical modified sentences were made by changing the accusative case particle (‘-*o*’) to the nominative case particle (‘*-ga*’).

Each modified sentence appeared only twice for each participant. Moreover, the same number of modified sentences were created for each pair of P^+^ and P^–^ sentences, and thus the use of words was perfectly counterbalanced across the four conditions of the original sentences. We were targeting the sentences and associated *structures*, not the words themselves in the present study; the repeated use of the same words would habituate any word-level processes, leaving out sentence-level processes under the conditions with the original sentences.

### Task

A syntactic decision task was performed, in which the participants decided whether the presented sentence was grammatically correct or not. This task was necessary to ensure the participants’ syntactic judgment based on the argument structure of each verb. In each trial, visual stimuli were presented in grey against a dark background (Figure 1B); the stimuli were projected from outside of the shield room onto a translucent screen (within a visual angle of 5.7°). For fixation, a red cross was always shown at the center of the screen. To inform the participants that the trial was beginning, a grey square was presented. Next, the stimuli of a sentence were presented in a serial, phrase-by-phrase manner. Each stimulus was presented for 300 ms, and the ISI was randomly varied from 300 to 400 ms. Lastly, a grey triangle was presented 700–800 ms after the verb onset to inform participants to start pushing one of two buttons according to the grammaticality of the sentence. The participants were required to respond within 1800 ms after the onset of the grey triangle. The task was performed in 10 separate MEG runs, each with 88 trials. The intertrial interval was randomly varied from 4700 to 5300 ms to reduce any periodical noises. The possessivity and canonicity of the sentences in each run were fully randomized and balanced. Stimulus presentation and behavioral data collection were controlled using the LabView software and interface (National Instruments, Austin, TX). Only trials with participants’ correct responses were used for analyzing reaction times (RTs) and MEG data.

### MEG Data Acquisition and Analyses

**Figure 2:**
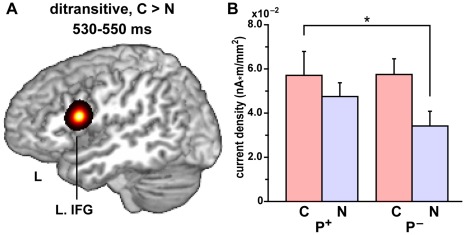
Significant activation with canonicity effects on ditransitive verbs. (A) Cortical activation showing a significant main effect of canonicity at 530–550 ms. A significant C > N effect (corrected *p*<0.05) was observed at a single cluster in the left (L.) IFG (shown in yellow to black), which was superimposed on a sagittal section of the standard brain at the peak [Talairach coordinates, (*x*, *y*, *z*) = (–48, 10, 18)]. (B) The current density in the left IFG cluster for each of the four conditions (mean ± SEM). An asterisk denotes the significant difference (*p*<0.05, paired *t*-test) between the two conditions, under which the same NP preceded a verb (see Table 2).

The raw MEG data were acquired with a 160-channel whole-head system (MEGvision; Yokogawa Electric Corporation, Kanazawa-city, Japan), and they were digitized with an on-line bandwidth of 0.3 Hz to 1000 Hz and a sampling rate of 2000 Hz. We basically followed the same procedures described in our previous study [33]. Using the BESA 5.2 software package (BESA GmbH, Munich, Germany), the MEG signals during the stimulus interval and the following shortest ISI (i.e., –100–700 ms for a verb, and –100–600 ms for a second NP; see Figure 1B) were analyzed, where the signals from –100 to 0 ms were used as a baseline (Figure 2A). Only artifact-free trials (peak-to-peak amplitude <2500 fT) with participants’ correct responses were averaged for each condition, and the averaged MEG signals were band-pass filtered in the frequency domain from 1 to 30 Hz to eliminate large eye movement noises. For mapping with the individual brain, high resolution T1-weighted MR images (repetition time, 30 ms; echo time, 8.0 ms; flip angle, 60°; field of view, 256×256 mm^2^; resolution, 1×1×1 mm^3^) were acquired using a 1.5-T Scanner (Stratis II, Premium; Hitachi Medical Corporation, Tokyo, Japan). The sensor positions for each of ten runs were realigned with five fiducial markers (small coils) on the head surface, and then coregistered with a least-squares fit algorithm to the MR images by attaching MR markers (alfacalcidol beads; diameter: 3 mm) at the same positions of fiducial markers (MEG Laboratory, Yokogawa Electric Corporation, Kanazawa-city, Japan). Using BrainVoyager QX 1.8 software (Brain Innovation, Maastricht, Netherlands), each individual brain was normalized to the image of the Montreal Neurological Institute (MNI) standard brain, which was already transformed into the Talairach space [44]. In order to perform a cortex-based data analysis, the gray and white matter of the transformed standard brain was segmented, and their boundary was then partitioned into 3445 cortical patches with a mean distance of 5.6 mm [45].

The distribution of cortical activation underlying the MEG signals was modeled with the minimum norm estimates (MNEs) of currents using BESA 5.2. A current dipole was perpendicularly placed at each center of the 3445 cortical patches, approximating any spatial distributions of currents on the cortex [46], [47]. The current density at each cortical patch was then obtained by dividing the strength of each current dipole by the mean area of the cortical patches. For each participant, the current densities at each cortical patch were averaged for a bin of 20 ms; this time bin was moved in 10 ms steps over the 100–700 ms period after the verb onset or the 100–600 ms period after the second NP onset. According to the sampling theorem, this sampling time of 20 ms corresponded to the highest frequency of 25 Hz, which was within the band-pass filter. We have successfully used the same time bin in our previous study [33].

We adopted whole-brain analyses that did not rely on any particular regions *a priori,* which is equivalent to performing all possible “functional region of interest (fROI)” analyses [48]. We compared the cortical responses under the four conditions with a cluster-based nonparametric test [49] as follows. First, we performed a two-way rANOVA (possessivity × canonicity) for the current density of each cortical patch, and selected all patches whose *F*-values were larger than the clustering threshold at *p*<0.0005. Next, we clustered the selected patches into connected sets on the basis of spatial adjacency (7 mm), and calculated cluster-level statistics by taking the sum of the *F*-values within a cluster as a representative index. The statistical results for each cluster were then spatially corrected for multiple comparisons across the whole brain (corrected *p*<0.05), using a permutation test for the current density of each condition [50], [51]. For example, in the comparison between the P^+^ and P^–^ sentences, the data for all cortical patches were exchanged between these two conditions in some of the 10 participants. For each permutation, the largest of the cluster-level statistics was determined among the clusters. There were 2^10^ = 1024 permutations, which produced a reference distribution of the cluster-level statistics for determining the corrected *P*-values. Correction for multiple comparisons using *F*-values is superior in sensitivity than that using simple mean differences of the current density [52]. Note that this method requires no assumption of a normal distribution or of the correlation structure of the data [50]. On each cortical patch in a cluster with significance, a 7-mm-diameter sphere was placed. Using SPM8 (http://www.fil.ion.ucl.ac.uk/spm/software/spm8) on MATLAB (http://www.mathworks.com/products/matlab), these spheres were spatially filtered with a Gaussian (full width of half maximum, 8 mm) and superimposed onto the standard brain with MRIcroN (http://www.cabiatl.com/mricro/mricron/index.html).

## Results

### Behavioral Data

Behavioral data on the accuracy and RTs for each condition of the original ditransitive sentences are shown in Table 4. A two-way rANOVA (possessivity × canonicity) for the accuracy and RTs showed no significant main effect of possessivity [accuracy: *F* (1, 9) = 0.01, *p* = 0.90; RTs: *F* (1, 9) = 0.61, *p* = 0.45] or canonicity [accuracy: *F* (1, 9) = 2.4, *p* = 0.16; RTs: *F* (1, 9) = 0.30, *p* = 0.60], with no significant interaction between these two factors [accuracy: *F* (1, 9) = 0.13, *p* = 0.73; RTs: *F* (1, 9) = 1.5, *p* = 0.25]. These behavioral results indicate that all of the four conditions were performed equally well by the participants. Therefore, selective responses among these conditions, if any, cannot be explained by performances alone.

**Table 4:** Behavioral data for ditransitive sentences under each condition.

Possessivity	Canonicity	Accuracy (%)	RTs (ms)
P^+^ sentences	Canonical	92.6±2.0	449±72
	Noncanonical	93.7±2.7	460±72
P^–^ sentences	Canonical	92.7±1.9	462±68
	Noncanonical	93.4±2.2	459±73
Mean		93.1±2.2	458±71

Data are shown as the mean ± SEM. Reaction times (RTs) were obtained from trials with correct responses.

We also compared the original sentences with the grammatical modified sentences (the mean ± SEM; accuracy: 89.9±3.2%; RTs: 481±72 ms) and the ungrammatical modified sentences (accuracy: 91.9±2.8%; RTs: 510±69 ms). A one-way rANOVA for the accuracy and RTs of these three types of sentences showed a significant main effect only for RTs [accuracy: *F* (2, 18) = 0.72, *p* = 0.50; RTs: *F* (2, 18) = 4.3, *p* = 0.03]. According to paired *t*-tests, the RTs of the original sentences were shorter than those of the grammatical and ungrammatical modified sentences [grammatical: *t* (9) = 2.4, *p* = 0.042; ungrammatical: *t* (9) = 2.4, *p* = 0.041]. It is possible that this difference was due to the higher frequency of the original sentences (400 per participant) than the grammatical and ungrammatical modified sentences (240 each). For the grammatical modified sentences, we also compared the canonical (accuracy: 90.0±3.3%; RTs: 473±68 ms) and noncanonical (accuracy: 89.7±3.2%; RTs: 489±78 ms) sentences, and there was no significant difference [accuracy: *t* (9) = 0.46, *p* = 0.66; RTs: *t* (9) = 0.72, *p* = 0.49], indicating that both conditions were also performed well by the participants.

### Canonicity Effects on Ditransitive Verbs

In the analyses of cortical responses (current density) to ditransitive verbs, we compared the activities of the whole brain under the four conditions, in which *phonologically same* verbs were presented (Table 2), using a statistical parametric map with a two-way rANOVA (possessivity × canonicity). At 530–550 ms after the verb onset, we found a significant main effect of canonicity at only one cluster of three adjacent cortical patches in the left IFG (corrected *p* = 0.048) [Talairach coordinates, (*x*, *y*, *z*) = (–43, 13, 16), (–48, 10, 18), and (–53, 10, 23); Brodmann’s areas (BAs) 44/45] (Figure 2A), which was confirmed to be C > N. In our paradigm (Figure 1A), both canonical P^+^ and noncanonical P^–^ sentences were with the DA order, and the accusative NP, i.e., the *second* NP, was physically identical (see Table 2). Even when the same NP preceded a verb, we found a significant C > N effect in the left IFG responses to the verb at 530–550 ms [*t* (9) = 3.4, *p* = 0.008] (Figure 2B). This predictive effect thus depended on *both* NPs that actually determined the canonicity of sentences. Indeed, the animacy of the first dative NP alone was not sufficient to determine the canonicity of sentences, because an animate NP could appear as a first NP in the *noncanonical* modified sentences (see Table 3). On the other hand, neither C > N effects at other time windows nor N > C effects over the 100–700 ms period were significant (corrected *p*>0.05). Moreover, neither a main effect of possessivity nor an interaction of the two factors was significant in the whole brain over the entire period.

We also performed an independent ROI analysis based on the left IFG activation at (–54, 9, 18) in MNI coordinates, which was previously identified with a past tense task using Japanese verbs [53]. This focus corresponded to (–51, 7, 18) in Talairach coordinates, and we defined a 7-mm-radius sphere at this voxel as a ROI (five patches). To *temporally* correct multiple comparisons across the whole time windows (100–700 ms after the verb onset with a bin of 20 ms), a permutation test was performed for the current density (clustering threshold at *p*<0.005). This additional analysis showed the significant main effect of canonicity at 530–560 ms (corrected *p* = 0.018), which was also C > N. Therefore, both whole-brain and ROI analyses suggest that the canonicity indeed modifies the brain activity in the left IFG.

Regarding the identical accusative NPs of the sentences with the DA order, i.e., the *second* NPs, there was no significant difference in the whole brain between canonical P^+^ and noncanonical P^–^ sentences over the 100–600 ms period after the second NP onset (corrected *p*>0.05). The canonicity effects shown above were thus selective to verbs. The activation patterns of the left IFG clearly established that the selective activations were due to the canonicity of ditransitive sentences, which was predictive in nature.

### Canonicity Effects on Monotransitive and Intransitive Verbs

**Figure 3:**
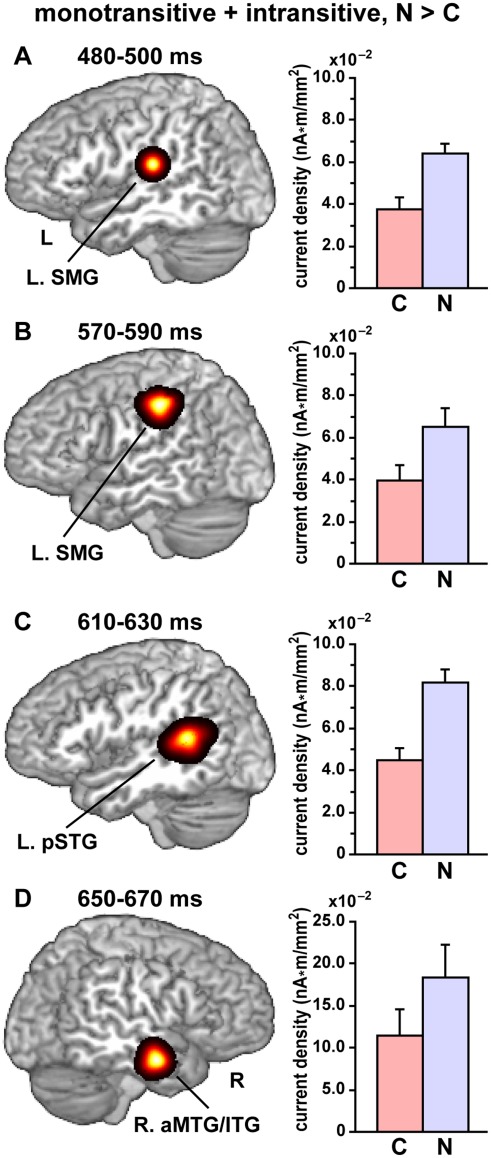
Significant activation with canonicity effects on monotransitive and intransitive verbs. We examined any canonicity effects for grammatical modified sentences with *monotransitive* or *intransitive* verbs. Each activation cluster was shown for a representative (i.e., with more activation) time bin of 20 ms, superimposed on a sagittal section of the standard brain at the peak. Paired *t*-tests resulted in a significant N > C effect (corrected *p*<0.05) in the following activated regions. The current density for canonical and noncanonical conditions is also shown for each cluster (mean ± SEM). (A) The left SMG activation [peak: (–50, –24, 7)] at 480–500 ms. (B) The left SMG activation [peak: (–57, –27, 11)] at 570–590 ms. (C) The left pSTG activation [peak: (–48, –45, 13)] at 610–630 ms. (D) The right (R.) aMTG/ITG activation [peak: (54, –3, –18)] at 650–670 ms.

We further examined any canonicity effects for grammatical modified sentences with *monotransitive* or *intransitive* verbs. As a prerequisite of canonicity effects, only cortical patches with a weak main effect of canonicity for *ditransitive* sentences at least in one time window at 100–700 ms (uncorrected *p*<0.05) were used. We used paired *t*-tests (factor: canonicity alone) with the same spatial correction procedure described above (corrected *p*<0.05). We observed a significant N > C effect on activations in the left supramarginal gyrus (SMG) at 480–510 ms (Figure 3A), as well as at 570–590 ms (Figure 3B). A significant N > C effect was also observed in the left pSTG at 600–650 ms (Figure 3C), as well as in the right anterior middle and inferior temporal gyrus (aMTG/ITG) at 650–670 ms (Figure 3D). There was no significant C > N effect in any cortical regions for the monotransitive and intransitive verbs, indicating that the C > N effect was more sensitive to ditransitive verbs.

## Discussion

In the present study, we found a significant main effect of canonicity on the current density in the left IFG at 530–550 ms (Figure 2). This effect was selective to canonical sentences with ditransitive verbs, i.e., C > N, and significant even when the precedent NP was physically identical. In addition, we observed a significant N > C effect for the modified sentences with monotransitive and intransitive verbs in the temporoparietal regions (Figure 3). These results demonstrate that the left IFG responses were selectively modulated by the canonicity of ditransitive sentences, in which the syntactic structures were different depending on the semantic contrasts between P^+^ and P^–^ sentences (Figure 1).

In our paradigm, possessivity and word orders were varied among the four conditions (Table 2), and thus at least two major factors other than the argument structures of the verb might have been involved. First, the sentence meanings were different between each pair of P^+^ and P^–^ sentences (Table 1), because different dative NPs were used and the verbs had different meanings. However, the main effect of possessivity was not significant in any regions or time windows. Second, the case particle of an NP just before the verb was different between DA and AD orders. However, the interaction between possessivity and canonicity, i.e., the effect of DA and AD orders (see Figure 1A), was not significant. Furthermore, our results cannot be explained by general cognitive factors, such as task difficulty, because there was no main effect of canonicity on the behavioral data. Linear order models for word sequences might be able to predict the upcoming word based on lexico-semantic association or statistics, i.e., transition probabilities between single words in a sentence [54], [55]. In the present experiment, however, we controlled statistical factors, such that the transition probabilities from the second NP, as well as from the two NPs, to the verb in ditransitive sentences (see the Materials and Methods). Therefore, any cortical responses modulated by the argument structures of a ditransitive verb depend on computations of syntactic structures that do not entirely reduce to linear orders or statistical effects.

As we have suggested that the left IFG is a grammar center [56], it is probable that the C > N effect observed in the present study was due to syntactic processes associated with the structural distance between the verb and NPs. More specifically, the shorter the structural distance between the verb and each NP was, the more influential the predictive effect of the two NPs became. During the syntactic decision task, the syntactic structure of each sentence would be constructed in an incremental manner based on the predicted argument structure of the ditransitive verb. We hypothesize that the argument structure predicted from both dative and accusative NPs was readily verified and processed further in a *canonical* sentence, where the NPs and verb were merged with a minimum structural distance, thus leading to the larger activations in the left IFG when the verb was presented. This hypothesis is consistent with the C > N effect in the left IFG responses, even when the same NP preceded a verb (Figure 2B). These results demonstrate that the left IFG plays a predictive role in syntactic processing, which depends on the canonicity determined by argument structures.

In the analyses of the responses to monotransitive and intransitive verbs, we found significant N > C effects in the temporoparietal regions (Figure 3). Structural computation of the modified sentences was simpler than that of ditransitive sentences, because there was no such a distinction as P^+^ or P^–^ sentences that affected the canonicity of sentences. It is thus more likely that the canonicity effects on monotransitive and intransitive verbs reflect non-syntactic (probably semantic) factors from the two NPs associated with the canonicity. At 480–510 and 570–590 ms, we observed a significant N > C effect in the left SMG, which may reflect the difficulty in processing lexical information for semantic-role assignment within noncanonical sentences. According to a cortical stimulation mapping study [57], the left SMG has been implicated in lexical retrieval during verb-naming. In our paradigm, a subject and another NP were scrambled in most noncanonical modified sentences (except monotransitive sentences with locative postposition ‘*-de*’, see Table 3). The N > C effect in the left pSTG at 600–650 ms was consistent with previous fMRI studies, which have contrasted noncanonical object-initial and canonical subject-initial sentences [3], [6]. We have previously reported that a focal region in the left pSTG/MTG was significantly activated by sentences containing syntactic or semantic anomalies [34]. The activation in the left pSTG may reflect reanalyses of anomalous or scrambled sentences, which are more confusing than canonical sentences. The N > C effect in the right aMTG/ITG at 650–670 ms was consistent with a transcranial magnetic stimulation (TMS) study with a synonym judgment task [58], suggesting that this effect may reflect the difficulty in processing semantic relationships between NPs for noncanonical sentences.

Some psycholinguistic studies have reported anticipatory or predictive effects of the semantic information from precedent phrases, using a plausibility judgment task or eye-tracking method [59], [60]. According to these views, semantic information of arguments can be incrementally integrated to accomplish on-line syntactic processing, such as filler-gap and semantic-role assignment. In our paradigm with P^+^ vs. P^–^ sentences, we suggest that more abstract semantic information of animacy or possessor, directly related to syntactic processing rather than the meaning of a word itself, was utilized as cues to make predictions. As demonstrated by the responses in the left IFG here, such predictions from precedent NPs would be formulated online and facilitate syntactic processing.

Our present results further indicate that predictive effects or top-down facilitation, which has been one of critical issues in the neuroscientific study of perception [61], [62], also plays an important role in syntactic processing. We also demonstrated that syntactic predictions generated in the left IFG actually depend on the structural distance between the NPs and verb, which has been emphasized in modern linguistics [41]. To conclude, the neuroimaging studies not only confirm the theory of linguistics but provide useful evidence for linguistics, auguring a future in which advances in the two fields are merged.
